# 1,3-Diiodo­azulene-2-carbonitrile

**DOI:** 10.1107/S1600536813008301

**Published:** 2013-04-05

**Authors:** Sebastian Förster, Wilhelm Seichter, Edwin Weber

**Affiliations:** a Institut für Organische Chemie, TU Bergakademie Freiberg, Leipziger Strasse 29, D-09596 Freiberg/Sachsen, Germany

## Abstract

In the title compound, C_11_H_5_I_2_N, the two iodine-atom substitutents with their large atomic sizes lead to short intra­molecular I⋯H distances (3.01 Å). In the crystal, the tris­ubstituted azulene system forms π-stacks [centroid–centroid distance = 3.6343 (11) Å] along the *a*-axis direction, showing the characteristic azulene inter­action mode between the electron-rich five-membered ring and the electron-deficient seven-membered ring. I⋯I [3.9129 (2) Å] non-covalent contacts are observed along with weak C—H⋯N and C—H⋯π. bonds.

## Related literature

For the naphthalene isomer azulene, see: Plattner & Pfau (1937 ▶). For the use of azulene derivatives for medical purposes, see: Shi *et al.* (2011 ▶). The synthesis of the title compound was performed starting from the azulene derivative 2-cyano­azulene (Nozoe *et al.*, 1962 ▶). For the synthesis of related compounds, see Schmitt *et al.* (1998 ▶); Suzuka & Yasunami (2008 ▶). For related structures, see: Förster *et al.* (2012 ▶); Hussain *et al.* (2005 ▶); Rahman *et al.* (2004 ▶). For halogen inter­actions in mol­ecular crystal structures, see: Awwadi *et al.* (2006 ▶); Metrangolo *et al.* (2008 ▶). For weak C—H⋯N hydrogen bonding, see: Desiraju & Steiner (1999 ▶). For C—H⋯π inter­actions, see: Nishio *et al.* (2009 ▶).
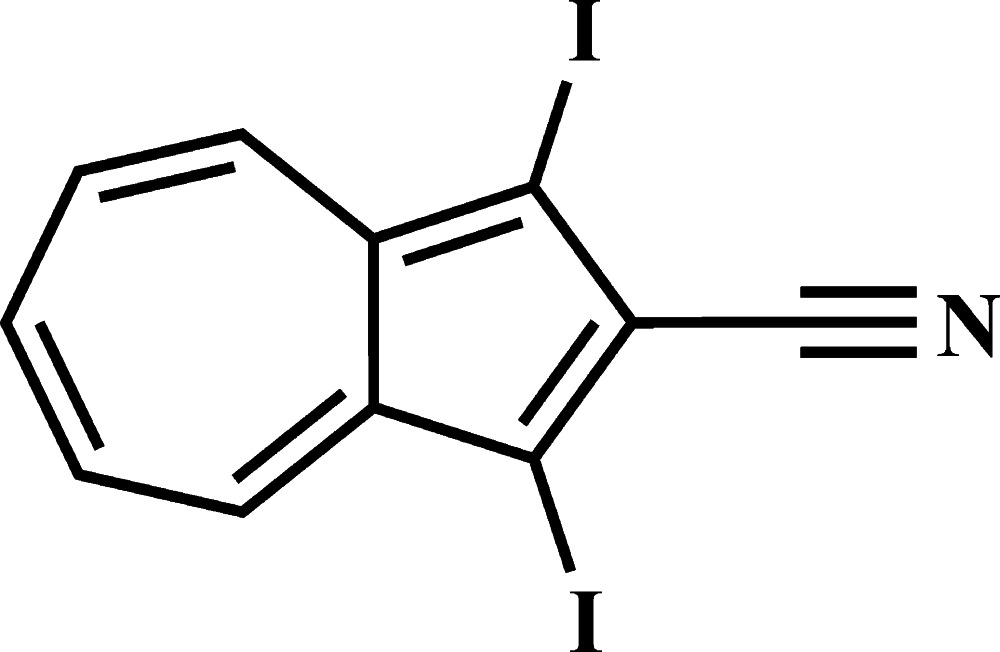


## Experimental

### 

#### Crystal data

C_11_H_5_I_2_N
*M* *_r_* = 404.96
Monoclinic, 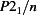

*a* = 4.2677 (1) Å
*b* = 14.9344 (4) Å
*c* = 16.7882 (4) Å
β = 96.952 (1)°
*V* = 1062.14 (5) Å^3^
*Z* = 4
Mo *K*α radiation
μ = 5.88 mm^−1^
*T* = 100 K
0.52 × 0.07 × 0.03 mm

#### Data collection

Bruker APEXII CCD area-detector diffractometer
Absorption correction: multi-scan (*SADABS*; Sheldrick, 2007) ▶ *T*_min_ = 0.150, *T*_max_ = 0.843
17691 measured reflections
4616 independent reflections
3863 reflections with *I* > 2σ(*I*)
*R*_int_ = 0.023

#### Refinement

*R*[*F* ^2^ > 2σ(*F* ^2^)] = 0.024
*wR*(*F* ^2^) = 0.058
*S* = 1.05
4616 reflections
127 parameters
H-atom parameters constrained
Δρ_max_ = 1.48 e Å^−3^
Δρ_min_ = −1.32 e Å^−3^

### 

Data collection: *APEX2* (Bruker, 2007 ▶); cell refinement: *SAINT* (Bruker, 2007 ▶); data reduction: *SAINT*; program(s) used to solve structure: *SHELXS97* (Sheldrick, 2008 ▶); program(s) used to refine structure: *SHELXL97* (Sheldrick, 2008 ▶); molecular graphics: *ORTEP-3 for Windows* (Farrugia, 2012 ▶); software used to prepare material for publication: *SHELXTL* (Sheldrick, 2008 ▶).

## Supplementary Material

Crystal structure: contains datablock(s) I, global. DOI: 10.1107/S1600536813008301/zp2002sup1.cif

Structure factors: contains datablock(s) I. DOI: 10.1107/S1600536813008301/zp2002Isup2.hkl

Supplementary material file. DOI: 10.1107/S1600536813008301/zp2002Isup3.cdx

Supplementary material file. DOI: 10.1107/S1600536813008301/zp2002Isup4.cml

Additional supplementary materials:  crystallographic information; 3D view; checkCIF report

## Figures and Tables

**Table 1:** Hydrogen-bond geometry (Å, °) *Cg*(C11—N1) is the mid-point of the C11—N1 bond.

*D*—H⋯*A*	*D*—H	H⋯*A*	*D*⋯*A*	*D*—H⋯*A*
C8—H8⋯N1^i^	0.95	2.62	3.400 (3)	139
C6—H6⋯*Cg*(C11—N1)^i^	0.95	2.76	3.63 (3)	152

Symmetry code: (i) 
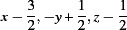
.

**Fig. 1.**
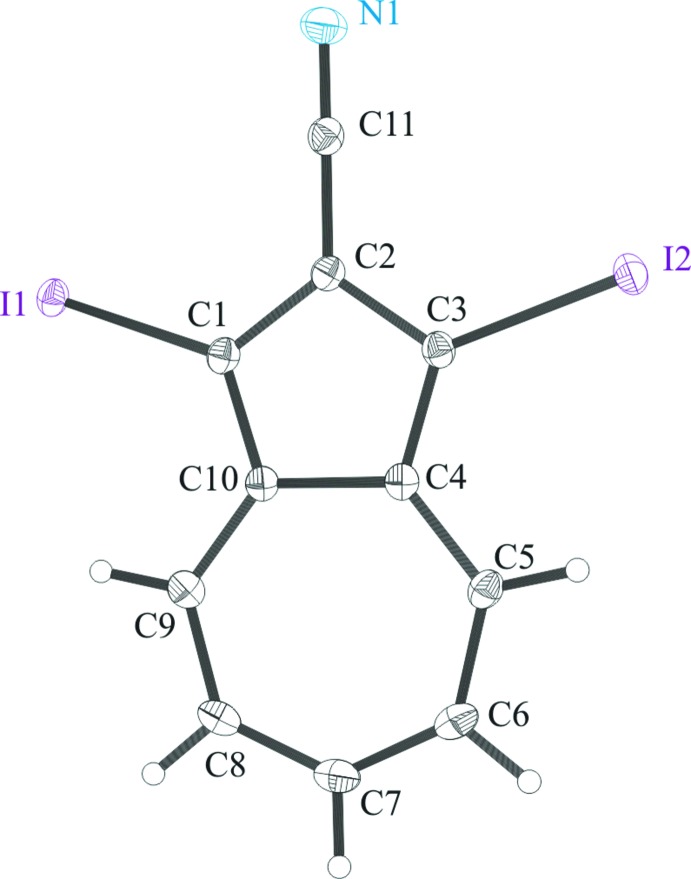
Asymmetric unit of the title compound, showing the atom numbering scheme. Displacement ellipsoids are drawn at the 50% probability level.

**Fig. 2.**
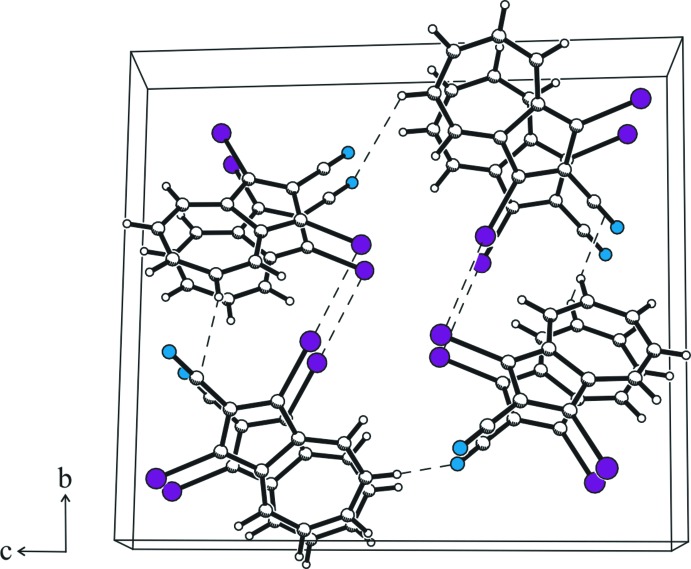
A view along the a axis of the crystal packing of the title compound. Intermolecular interactions are represented as dashed lines.

## supplementary crystallographic information

### Comment

The naphthalene isomer azulene is a well known blue nonbenzenoid aromatic hydrocarbon (Plattner 1937). Besides the use of azulene derivatives for medical purposes (Shi 2011), special electronic properties and redox behaviour makes them interesting compounds for electronic applications (Förster *et al.* 2012). In this regard, the present trisubstituted azulene derivative is a promising intermediate. In the crystal structure of this compound, the asymmetric unit contains one molecule featuring an almost planar azulene ring system with a maximum deviations of 0.023 (2) and 0.023 (2) Å (Fig.1). Short intramolecular distances (3.01 Å) involving the iodine atoms and their neighboring H atoms (I1···H9, I2···H5) are a further characteristic of the molecular structur. Along the crystallographic *a*-axis, the crystal structure is stabilized by formation of molecular stacks with a distance of 3.46 Å between the planes of the molecules. The parallel orientated azulene ring systems are arranged in an offset *face-to-face* fashion showing π···π overlap between the five- and seven-membered ring components in conformity with their dipole character. In direction of the crystallographic *b* and *c* axes, these stacks are connected *via* C—H···N contacts [C8—H8···N1 (3/2+x,1/2-y,1/2+z) 2.62 Å, 139.1°] (Desiraju & Steiner, 1999), I···I interactions [I1···I2 (3/2-x,-1/2+y,1/2-z) 3.91 Å, 91°] (Metrangolo *et al.*, 2008) and C—H···π contacts [C6—H6···center(C11, N1) (1/2-x,-1/2+y,1/2-z) 2.76 Å, 152.6°] (Nishio *et al.*, 2009) (Fig 2).

### Experimental

The synthesis of the title compound was done starting from the literature known azulene derivative 2-cyanoazulene (Nozoe *et al.*, 1962). This latter compound (0.1 g, 0.65 mmol) and *N*-iodosuccinimide (0.44 g, 1.96 mmol) were dissolved in 20 ml dichloromethane. The solution was stirred for 8 h under reflux. After removal of the solvent, the residue was purified by column chromatography on SiO_2_ [60 F_254_ Merck eluent: hexane/ethyl acetate (4:1)] to yield 0.24 g (91%) product as a green solid. Analytical data: mp = 211°C; ^1^H-NMR: (CDCl_3_) δ/p.p.m. = 7.46 (t, 2 H, ArH, ^3^J_HH_ = 9.78 Hz), 7.83 (t, 1 H, ArH, ^3^J_HH_ = 9.85 Hz), 8.30 (d, 2 H, ArH, ^3^J_HH_ = 9.95 Hz); ^13^C-NMR: (CDCl_3_) δ/p.p.m. = 78.41(ArC), 117.30(CN), 126.86(ArC), 129.23(ArC), 141.00(ArC), 142.82(ArC), 143.06(ArC); GC/MS calc.: 405; found: 405 [*M*]^+.^. Crystallization by slow evaporation from *n*-hexane yielded suitable crystals.

### Refinement

Aromatic H atoms were positioned geometrically and allowed to ride on their respective parent atoms, with C—H = 0.95 Å and *U*_iso _= 1.2 *U*_eq_(C).

### Crystal data

**Table:**

C_11_H_5_I_2_N	*F*(000) = 736
*M**_r_* = 404.96	*D*_x_ = 2.532 Mg m^−^^3^
Monoclinic, *P*2_1_/*n*	Mo *K*α radiation, λ = 0.71073 Å
Hall symbol: -P 2yn	Cell parameters from 7883 reflections
*a* = 4.2677 (1) Å	θ = 2.7–36.5°
*b* = 14.9344 (4) Å	µ = 5.88 mm^−^^1^
*c* = 16.7882 (4) Å	*T* = 100 K
β = 96.952 (1)°	Needle, green
*V* = 1062.14 (5) Å^3^	0.52 × 0.07 × 0.03 mm
*Z* = 4	

### Data collection

**Table:**

Bruker APEXII CCD area-detector diffractometer	4616 independent reflections
Radiation source: fine-focus sealed tube	3863 reflections with *I* > 2σ(*I*)
Graphite monochromator	*R*_int_ = 0.023
phi and ω scans	θ_max_ = 35.1°, θ_min_ = 1.8°
Absorption correction: multi-scan (*SADABS*; Sheldrick, 2007)	*h* = −6→4
*T*_min_ = 0.150, *T*_max_ = 0.843	*k* = −24→22
17691 measured reflections	*l* = −24→27

### Refinement

**Table:**

Refinement on *F*^2^	Primary atom site location: structure-invariant direct methods
Least-squares matrix: full	Secondary atom site location: difference Fourier map
*R*[*F*^2^ > 2σ(*F*^2^)] = 0.024	Hydrogen site location: difference Fourier map
*wR*(*F*^2^) = 0.058	H-atom parameters constrained
*S* = 1.05	*w* = 1/[σ^2^(*F*_o_^2^) + (0.0289*P*)^2^ + 0.3529*P*] where *P* = (*F*_o_^2^ + 2*F*_c_^2^)/3
4616 reflections	(Δ/σ)_max_ = 0.001
127 parameters	Δρ_max_ = 1.48 e Å^−^^3^
0 restraints	Δρ_min_ = −1.32 e Å^−^^3^

### Special details

**Table:**

Geometry. All s.u.'s (except the s.u. in the dihedral angle between two l.s. planes) are estimated using the full covariance matrix. The cell s.u.'s are taken into account individually in the estimation of s.u.'s in distances, angles and torsion angles; correlations between s.u.'s in cell parameters are only used when they are defined by crystal symmetry. An approximate (isotropic) treatment of cell s.u.'s is used for estimating s.u.'s involving l.s. planes.
Refinement. Refinement of *F*^2^ against ALL reflections. The weighted *R*-factor *wR* and goodness of fit *S* are based on *F*^2^, conventional *R*-factors *R* are based on *F*, with *F* set to zero for negative *F*^2^. The threshold expression of *F*^2^ > 2σ(*F*^2^) is used only for calculating *R*-factors(gt) *etc*. and is not relevant to the choice of reflections for refinement. *R*-factors based on *F*^2^ are statistically about twice as large as those based on *F*, and *R*- factors based on ALL data will be even larger.

### Fractional atomic coordinates and isotropic or equivalent isotropic displacement parameters (Å^2^)

**Table:**

	*x*	*y*	*z*	*U*_iso_*/*U*_eq_	
I1	0.78365 (3)	0.118650 (8)	0.153674 (8)	0.01453 (4)	
I2	0.54859 (3)	0.379652 (8)	0.426773 (8)	0.01708 (4)	
N1	1.0480 (4)	0.14850 (13)	0.39516 (11)	0.0216 (4)	
C1	0.6331 (4)	0.23056 (12)	0.21157 (11)	0.0131 (3)	
C2	0.7006 (4)	0.24765 (12)	0.29426 (11)	0.0129 (3)	
C3	0.5399 (4)	0.32630 (12)	0.31275 (11)	0.0131 (3)	
C4	0.3685 (4)	0.35906 (12)	0.24230 (11)	0.0126 (3)	
C10	0.4300 (4)	0.29734 (12)	0.17661 (11)	0.0126 (3)	
C9	0.3126 (5)	0.30350 (13)	0.09587 (12)	0.0157 (3)	
H9	0.3852	0.2591	0.0620	0.019*	
C8	0.1030 (5)	0.36596 (14)	0.05796 (12)	0.0182 (4)	
H8	0.0509	0.3581	0.0018	0.022*	
C7	−0.0398 (5)	0.43785 (14)	0.09082 (13)	0.0186 (4)	
H7	−0.1803	0.4714	0.0540	0.022*	
C6	−0.0090 (5)	0.46858 (13)	0.16964 (13)	0.0178 (4)	
H6	−0.1279	0.5206	0.1788	0.021*	
C5	0.1722 (4)	0.43354 (12)	0.23683 (12)	0.0148 (3)	
H5	0.1601	0.4648	0.2856	0.018*	
C11	0.8938 (5)	0.19344 (13)	0.35015 (12)	0.0151 (3)	

### Atomic displacement parameters (Å^2^)

**Table:**

	*U*^11^	*U*^22^	*U*^33^	*U*^12^	*U*^13^	*U*^23^
I1	0.01660 (6)	0.01155 (6)	0.01611 (6)	0.00045 (4)	0.00466 (4)	−0.00113 (4)
I2	0.02230 (7)	0.01668 (6)	0.01229 (6)	0.00117 (4)	0.00219 (4)	−0.00198 (4)
N1	0.0239 (9)	0.0197 (8)	0.0198 (9)	0.0022 (7)	−0.0036 (7)	0.0007 (7)
C1	0.0144 (7)	0.0101 (7)	0.0152 (8)	−0.0011 (6)	0.0029 (6)	−0.0011 (6)
C2	0.0131 (7)	0.0110 (7)	0.0145 (8)	−0.0006 (6)	0.0020 (6)	0.0001 (6)
C3	0.0150 (8)	0.0117 (7)	0.0127 (8)	−0.0010 (6)	0.0022 (6)	−0.0011 (6)
C4	0.0131 (7)	0.0113 (7)	0.0136 (8)	−0.0022 (6)	0.0027 (6)	−0.0001 (6)
C10	0.0137 (7)	0.0109 (7)	0.0134 (8)	−0.0013 (6)	0.0026 (6)	−0.0001 (6)
C9	0.0177 (8)	0.0147 (8)	0.0146 (8)	−0.0023 (6)	0.0017 (6)	−0.0003 (6)
C8	0.0200 (9)	0.0204 (9)	0.0133 (8)	−0.0017 (7)	−0.0008 (7)	0.0031 (7)
C7	0.0161 (8)	0.0212 (9)	0.0181 (9)	0.0006 (7)	0.0004 (7)	0.0067 (7)
C6	0.0168 (8)	0.0132 (8)	0.0234 (10)	0.0024 (6)	0.0026 (7)	0.0033 (7)
C5	0.0156 (8)	0.0116 (7)	0.0178 (9)	0.0002 (6)	0.0044 (7)	−0.0001 (6)
C11	0.0161 (8)	0.0129 (8)	0.0162 (8)	−0.0009 (6)	0.0017 (6)	−0.0019 (6)

### Geometric parameters (Å, º)

**Table:**

I1—C1	2.0741 (18)	C10—C9	1.390 (3)
I2—C3	2.0697 (19)	C9—C8	1.392 (3)
N1—C11	1.155 (3)	C9—H9	0.9500
C1—C10	1.403 (3)	C8—C7	1.382 (3)
C1—C2	1.407 (3)	C8—H8	0.9500
C2—C3	1.413 (3)	C7—C6	1.392 (3)
C2—C11	1.424 (3)	C7—H7	0.9500
C3—C4	1.401 (3)	C6—C5	1.390 (3)
C4—C5	1.389 (3)	C6—H6	0.9500
C4—C10	1.485 (3)	C5—H5	0.9500
			
C10—C1—C2	109.05 (16)	C10—C9—H9	115.6
C10—C1—I1	125.99 (14)	C8—C9—H9	115.6
C2—C1—I1	124.80 (13)	C7—C8—C9	128.91 (19)
C1—C2—C3	108.70 (16)	C7—C8—H8	115.5
C1—C2—C11	125.46 (17)	C9—C8—H8	115.5
C3—C2—C11	125.83 (17)	C8—C7—C6	129.82 (19)
C4—C3—C2	108.81 (16)	C8—C7—H7	115.1
C4—C3—I2	126.53 (14)	C6—C7—H7	115.1
C2—C3—I2	124.62 (13)	C5—C6—C7	128.80 (19)
C5—C4—C3	125.77 (18)	C5—C6—H6	115.6
C5—C4—C10	127.40 (17)	C7—C6—H6	115.6
C3—C4—C10	106.80 (16)	C4—C5—C6	128.82 (19)
C9—C10—C1	125.92 (18)	C4—C5—H5	115.6
C9—C10—C4	127.44 (17)	C6—C5—H5	115.6
C1—C10—C4	106.64 (16)	N1—C11—C2	179.1 (2)
C10—C9—C8	128.76 (19)		
			
C10—C1—C2—C3	−0.1 (2)	I1—C1—C10—C4	175.27 (13)
I1—C1—C2—C3	−175.66 (13)	C5—C4—C10—C9	2.5 (3)
C10—C1—C2—C11	178.71 (18)	C3—C4—C10—C9	−179.32 (18)
I1—C1—C2—C11	3.2 (3)	C5—C4—C10—C1	−177.67 (18)
C1—C2—C3—C4	0.4 (2)	C3—C4—C10—C1	0.5 (2)
C11—C2—C3—C4	−178.40 (18)	C1—C10—C9—C8	177.6 (2)
C1—C2—C3—I2	178.31 (13)	C4—C10—C9—C8	−2.6 (3)
C11—C2—C3—I2	−0.5 (3)	C10—C9—C8—C7	0.4 (4)
C2—C3—C4—C5	177.63 (18)	C9—C8—C7—C6	1.5 (4)
I2—C3—C4—C5	−0.2 (3)	C8—C7—C6—C5	−1.1 (4)
C2—C3—C4—C10	−0.5 (2)	C3—C4—C5—C6	−178.5 (2)
I2—C3—C4—C10	−178.39 (13)	C10—C4—C5—C6	−0.8 (3)
C2—C1—C10—C9	179.57 (18)	C7—C6—C5—C4	0.1 (4)
I1—C1—C10—C9	−4.9 (3)	C1—C2—C11—N1	−55 (15)
C2—C1—C10—C4	−0.2 (2)	C3—C2—C11—N1	123 (15)

### Hydrogen-bond geometry (Å, º)

Cg(C11—N1) is the mid-point of the C11—N1 bond.

**Table:**

*D*—H···*A*	*D*—H	H···*A*	*D*···*A*	*D*—H···*A*
C8—H8···N1^i^	0.95	2.62	3.400 (3)	139
C6—H6···*Cg*(C11—N1)^i^	0.95	2.76	3.63 (3)	152

Symmetry code: (i) *x*−3/2, −*y*+1/2, *z*−1/2.
